# Combining phospholipases and a liquid lipase for one-step biodiesel production using crude oils

**DOI:** 10.1186/1754-6834-7-29

**Published:** 2014-02-26

**Authors:** Silvia Cesarini, Rune Falkenberg Haller, Pilar Diaz, Per Munk Nielsen

**Affiliations:** 1 Department of Microbiology, University of Barcelona, Avenida Diagonal 643, Barcelona 08028, Spain; 2 Department of Analytical Development, Novozymes A/S, Hallas Alle 1, Kalundborg 4400, Denmark; 3 Department of Bioenergy Opportunities, Novozymes A/S, Krogshoejvej 36, Bagsvaerd 2880, Denmark

**Keywords:** Enzymatic biodiesel, Liquid lipase, Crude oils, Phospholipases

## Abstract

**Background:**

Enzymatic biodiesel is becoming an increasingly popular topic in bioenergy literature because of its potential to overcome the problems posed by chemical processes. However, the high cost of the enzymatic process still remains the main drawback for its industrial application, mostly because of the high price of refined oils. Unfortunately, low cost substrates, such as crude soybean oil, often release a product that hardly accomplishes the final required biodiesel specifications and need an additional pretreatment for gums removal. In order to reduce costs and to make the enzymatic process more efficient, we developed an innovative system for enzymatic biodiesel production involving a combination of a lipase and two phospholipases. This allows performing the enzymatic degumming and transesterification in a single step, using crude soybean oil as feedstock, and converting part of the phospholipids into biodiesel. Since the two processes have never been studied together, an accurate analysis of the different reaction components and conditions was carried out.

**Results:**

Crude soybean oil, used as low cost feedstock, is characterized by a high content of phospholipids (900 ppm of phosphorus). However, after the combined activity of different phospholipases and liquid lipase Callera Trans L, a complete transformation into fatty acid methyl esters (FAMEs >95%) and a good reduction of phosphorus (P <5 ppm) was achieved. The combination of enzymes allowed avoidance of the acid treatment required for gums removal, the consequent caustic neutralization, and the high temperature commonly used in degumming systems, making the overall process more eco-friendly and with higher yield. Once the conditions were established, the process was also tested with different vegetable oils with variable phosphorus contents.

**Conclusions:**

Use of liquid lipase Callera Trans L in biodiesel production can provide numerous and sustainable benefits. Besides reducing the costs derived from enzyme immobilization, the lipase can be used in combination with other enzymes such as phospholipases for gums removal, thus allowing the use of much cheaper, non-refined oils. The possibility to perform degumming and transesterification in a single tank involves a great efficiency increase in the new era of enzymatic biodiesel production at industrial scale.

## Background

Lipase-catalyzed biodiesel production is an intensive area of research because of its potential to generate eco-friendly fuel. Indeed, employment of lipases as biocatalysts in the transesterification of triacylglycerides allows mild reaction conditions and easy recovery of glycerol, without need for further purification or chemical waste production. In addition, the enzymatic process tolerates the common water content of oil and increases the biodiesel yield, avoiding the typical soap formation due to alkaline transesterification [1]. Various lipases from different sources, generally in their immobilized form, have been investigated for transesterification using several substrates [2]. To date, high lipase activity and stability, with very good conversion rates in short reaction times, have been reported [3-6]. However, the high costs of enzyme immobilization remain a major drawback for lipase-catalyzed biodiesel production. The immobilization process and the supports used make the enzymatic transesterification economically not competitive against the chemical process [3,7]. According to Ghaly *et al*. (2010), the cost of the enzyme makes up to 90% of the total cost of the enzymatic process [3]. Moreover, lipase inhibition due to binding of the released glycerol to the supports has been reported [8]. In order to make the process more competitive and sustainable, the possibility of using soluble, non-immobilized lipases in biodiesel production is being investigated nowadays [9-11]. Soluble lipases can also be used under mild reaction conditions, have a faster reaction time, display higher conversion rates than immobilized enzymes [9], and allow, without any fatty acid methyl esters (FAMEs) yield loss, the presence of water in the process, which is required for oil pretreatment or during downstream steps [11]. Furthermore, liquid lipases can be produced and sold at a much lower price [12,13], and can also be re-used after recovery from the glycerin phase. For instance, Callera Trans L maintained up to 95% activity after recycling (AR Madsen, personal communication). But in contrast with immobilized lipases, the necessary number of re-uses of liquid lipases could be much lower due to the large difference in price of both types of enzymes. The use of liquid instead of immobilized enzyme has resulted in a significant simplification of oil transesterification and is the background for the new, cost-effective process developed by Novozymes (Bagsværd, Denmark) using liquid lipase Callera Trans L, which is now being scaled-up at several production plants for biodiesel synthesis from different feedstocks [14].

Nowadays, biodiesel is mainly produced by transesterification of edible oils such as those from soybean, rapeseed, sunflower, or palm [15]. The cost of feedstocks is another important economical factor in biodiesel production, which indicates that selection of the appropriate raw material is of major importance for ensuring the feasibility of the process at industrial scale [16]. Thus, exploring the use of low value or non-edible feedstocks is a goal for biodiesel producing partners. In this context, non-refined (crude) soybean oil is an alternative solution already investigated for enzymatic transesterification [6,11,17]. However, crude, non-degummed oils contain impurities as phosphorous compounds (phospholipids), which have to be removed for efficient transesterification. Crude soybean oil generally has a phosphorus (gums) content of 800 to 1,200 ppm, equivalent to 2 to 3% of phospholipids [18], which can cause problems during storage due to precipitate formation and water accumulation. Moreover, according to the legal specifications, phosphorus concentration in final biodiesel must be reduced to less than 10 ppm (EN14214:2003; ASTM D6751:2012). For biodiesel production, a high content of phospholipids in the raw feedstocks means a concomitant loss of yield in FAMEs production, as the fatty acids enclosed into the phospholipid molecules are not accessible to the lipase for transesterification. Phospholipids are commonly removed by previously developed refining/degumming processes of physical or chemical nature. In the 1990s the first industrial enzymatic oil-degumming process was launched with EnzyMax® (Lurgi AG, Frankfurt, Germany), based on phospholipase conversion of non-hydratable phospholipids (NHPs) to hydratable lyso-phospholipids [19]. NHPs, which consist of phospholipids combined with metal cations, such as Ca^2+^ or Mg^2+^, can be chemo-enzymatically converted into hydratable compounds and simply removed by water washing and centrifugation [20]. The easily hydratable phospholipids are phosphocholine (PC), phosphatidylinositol (PI), and lysophosphatidylcholine (LPC), whereas phosphatidylethanolamine (PE) and phosphatidic acid (PA) are generally considered as NHPs [21]. A guideline for industrial enzymatic degumming was reported by Cowan and Nielsen in 2008, suggesting a first step of citric acid treatment, where the acid is distributed with a high-shear mixer in the oil at high temperatures, to chelate the metals and to open the phospholipid micelles; following the citric acid treatment, the oil is cooled down to the optimal temperature for a final phospholipase treatment. Water (up to 1.5 to 2.5%) is added together with the enzyme and sodium hydroxide, and the enzymatic reaction is carried out for 2 to 6 h [18].

Different types of microbial phospholipases have been described depending on the acyl ester bond they hydrolyze in the phospholipid molecule [22], which could be applied to enzymatic degumming. Phospholipase A_1_ (PLA_1_), which catalyzes the hydrolysis of the fatty acid at position 1 in the phospholipid molecule, releasing a lyso-phospholipid and a free fatty acid (FFA), has already been applied to the degumming process [18,22,23] also in combination with phospholipase C (PLC), which cleaves the phosphorus–oxygen bond between glycerol and phosphate, releasing a diacylglycerol (DAG) and the phosphate ester group [24].

To search for a more sustainable, economic, and competitive process for biodiesel production, in this work we tested two phospholipases (PLA_1_ and PLC), a lyso-phospholipase (LLPL-2), and their combinations, with the previously reported liquid lipase Callera Trans L in order to unify the enzymatic oil degumming with the transesterification, aimed at producing a final biodiesel that meets the phosphorus legal limits. Degumming and transesterification processes were studied combined or separately to define the optimal conditions for each enzyme applied (summarized in Table 1), and two main parameters were studied: the effect of methanol, necessary for the transesterification, on phospholipases, and the effect of citric acid, usually used as helper in oil degumming, on lipase activity. Crude soybean oil was used to set up the general conditions of the process, which was additionally tested on other difficult raw materials to analyze possible drawbacks.

**Table 1:** Properties and working conditions in transesterification and degumming of all enzymes used

	**Transesterification**	**Enzymatic degumming**		
**Enzyme**	**Lipase**	**Phospholipase A**_**1**_	**Phospholipase C**	**Lyso-phospholipase**
**Commercial name**	**Callera Trans® L (Novozymes A/S)**	**Lecitase® Ultra (Novozymes A/S)**	**Purifine® (Verenium)**	**LLPL-2 (Novozymes A/S)**
pH	5	4.5 to 5.5	7	4.5 to 4.8
Temperature	35°C	50 to 55°C	60°C	40 to 45°C
Time reaction	24 h	4 to 6 h	2 h	
Dosage	1%	30 ppm	200 ppm	250 to 500 ppm
H_2_O required	2 to 3.5%	3%	1 to 4%	
Methanol^a^	1.5 eqs			
Acid pretreatment^b^		0.065% Citric acid/0.025% Phosphoric acid		
Caustic neutralization^b^		2 eqs NaOH		

^a^Required for transesterification, never studied in enzymatic degumming; ^b^generally used to help the enzymatic degumming, unknown effect on lipase activity.

## Results and discussion

### Citric acid effect on lipase activity

**Figure 1:**
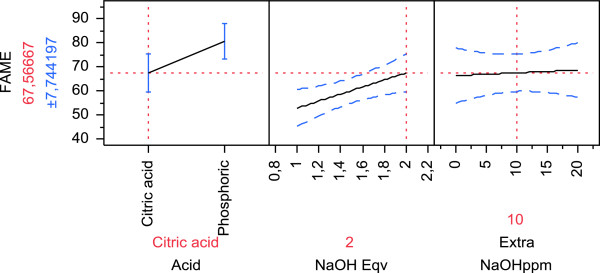
**Prediction profile for citric acid obtained from RSM analysis.** Graphical plotting of the effect of citric/phosphoric acid, pH, and extra NaOH on FAME production, made with JMP software (SAS Institute Inc.) (Rs = 0.88). FAME, fatty acid methyl ester; RSM, response surface methodology.

Citric acid, due to its chelating action towards Ca^2+^ and Mg^2+^ ions, is used as helper in degumming for disruption of phospholipid micelles [18,23]. Aimed at combining degumming and transesterification in the same pot, the influence of citric acid in the reaction system was a determining parameter studied here. A main point of interest was to investigate how citric acid might affect transesterification and lipase activity, in relation to pH variations. Accordingly, the pH effect on FAMEs production was analyzed by response surface methodology (RSM), by adding different amounts of NaOH to the reaction mixture, according to the patterns described in Table 2, which also includes the experimental response obtained for FAMEs production. Figure 1 shows the prediction profile for FAMEs content after citric acid use in soybean oil, obtained from the data analysis performed with JMP software (SAS Institute Inc., Cary, NC, USA). A clear negative effect of citric acid on transesterification, determined as a significant value (prob > |t| = <0.0001), was observed (Figure 1, left plot). Maximum production with citric acid was achieved when 2 eqs of NaOH were added for neutralization, but only 67% FAMEs release was obtained under such conditions. The central plot in Figure 1 shows the effect of pH controlled by caustic neutralization: using 2 eqs of NaOH (corresponding to 270 ppm) to balance the effect of citric acid, resulting in a pH of 5 (D Cowan, personal communication), and optimal conditions for lipase Callera Trans L, a good biodiesel release should be achieved. However, despite the slight increase of the percentage of FAMEs with the increase of added NaOH, still a too low biodiesel production was reached. The right plot in Figure 1 shows that 10 or 20 extra ppm of NaOH, generally used with some difficult raw materials for a more efficient biodiesel production, had no effect on transesterification. Even though FAMEs production with phosphoric acid was higher than with citric acid (Figure 1, left plot), transesterification was not complete, as it has been described that Callera Trans L, used under optimum reaction conditions, can reach >95% transformation [11]. Moreover, use of phosphoric acid in an industrial biodiesel production plant could be a problem due to its toxicity; for this reason citric acid is nowadays being used in industrial degumming processes. As shown above (Figure 1), the effect of pH on the enzyme was not strongly significant, suggesting that the lipase inhibition effect observed when phosphoric acid was used could be due to a too strong chelating effect that might remove metal ions from the enzyme molecule, necessary for tertiary structure folding. Otherwise, a direct competition of the acid molecules with the substrate for the catalytic site of the enzyme could be hypothesized. Given the limited FAMEs production obtained when an acid was present in the system, in the following combined degumming/transesterification process, we decided to avoid the acid treatment, opting out for a good transesterification rate instead of an optimum content of phosphorus in the final biodiesel.

**Table 2:** Experimental plan and results (FAMEs release) from testing acid, NaOH addition, and NaOH excess additions

**Pattern**	**Variables**			**Response**
	**X1 (acid)**	**NaOH (eqv)**	**Extra NaOH (ppm)**	**FAMEs (%)**
212	Phosphoric acid	1	10	84.3
132	Citric acid	2	10	67.8
222	Phosphoric acid	1.5	10	83.9
131	Citric acid	2	0	67.1
232	Phosphoric acid	2	10	84.7
211	Phosphoric acid	1	0	78.8
231	Phosphoric acid	2	0	77.6
112	Citric acid	1	10	67.0
121	Citric acid	1.5	0	77.3
221	Phosphoric acid	1.5	0	79.5
122	Citric acid	1.5	10	53.6
213	Phosphoric acid	1	20	91.1
113	Citric acid	1	20	51.3
111	Citric acid	1	0	43.5
133	Citric acid	2	20	71.1
123	Citric acid	1.5	20	82.2
223	Phosphoric acid	1.5	20	85.6
233	Phosphoric acid	2	20	79.5

### Effect of methanol on degumming

The second main parameter to be investigated for the combined degumming and transesterification reaction was the possible phospholipase inhibition due to the presence of methanol in the reaction mixture. In order to preserve the stability of the lipase, methanol was pumped into the reaction mixture following a slow continuous gradient of 0.4 ml/h during 10 h, for a total amount of 1.5 molar equivalents of methanol to the total fatty acids (in glycerides and FFAs) in the oil. Taking into consideration that during an efficient transesterification reaction methanol is consumed by the lipase to form methyl esters, this compound is never present in the reaction mixture at the amount of 1.5 eqs. Knowing that 1 eq of methanol is required for a theoretical complete transesterification, the maximum amount of methanol left in the system that might inhibit phospholipases, would be 0.5 eqs.

To investigate the effect of methanol on phospholipases, a normal degumming process, including the conventional citric acid step, on soybean oil was carried out with addition of 0.5 eqs methanol during 10 h. Reactions were incubated for 24 h at 35°C, simulating a combination of the two processes. All phospholipases and the corresponding control samples were tested in the same trials with and without methanol, as specified in Table 3, and the final phosphorous content was determined. To avoid any artifacts due to the recovery method upon phospholipids precipitation, samples were analyzed both directly from the stopped reaction (data not shown) and after methanol evaporation (Table 3). Final phosphorus content was measured by inductively coupled plasma optical emission spectrometry (ICP-OES) and expressed in ppm. For the two recovery conditions used, higher phosphorus values were found in the presence of methanol for all enzyme mixtures tested. Higher phosphorus amounts appeared as well after incubation of the original raw material with methanol, suggesting a direct action of methanol on the phospholipids from soybean oil and excluding any kind of phospholipase inhibition due to methanol.

**Table 3:** Phosphorus content of samples treated or untreated with methanol

**Condition**	**Without MeOH**	**With MeOH**
Raw oil^a^	64	575
AD^b^	<5	18
AD + PLA_1_^c^	<5	5
AD + PLC^c^	<5	7
AD + PLA_1_ + PLC^c^	<5	<5
AD + PLA_1_ + LLPL-2^c^	<5	14

^a^Soybean oil with approximately 900 ppm P; ^b^AD batch where only the acid degumming step followed by caustic neutralization was performed; ^c^complete degumming: acid treatment, caustic neutralization, and enzymatic reactions with different phospholipases. Phosphorus content expressed in ppm. AD, acid degumming; PLA_1_, phospholipase A_1_; PLC, phospholipase C; LLPL-2, lyso-phospholipase.

**Figure 2:**
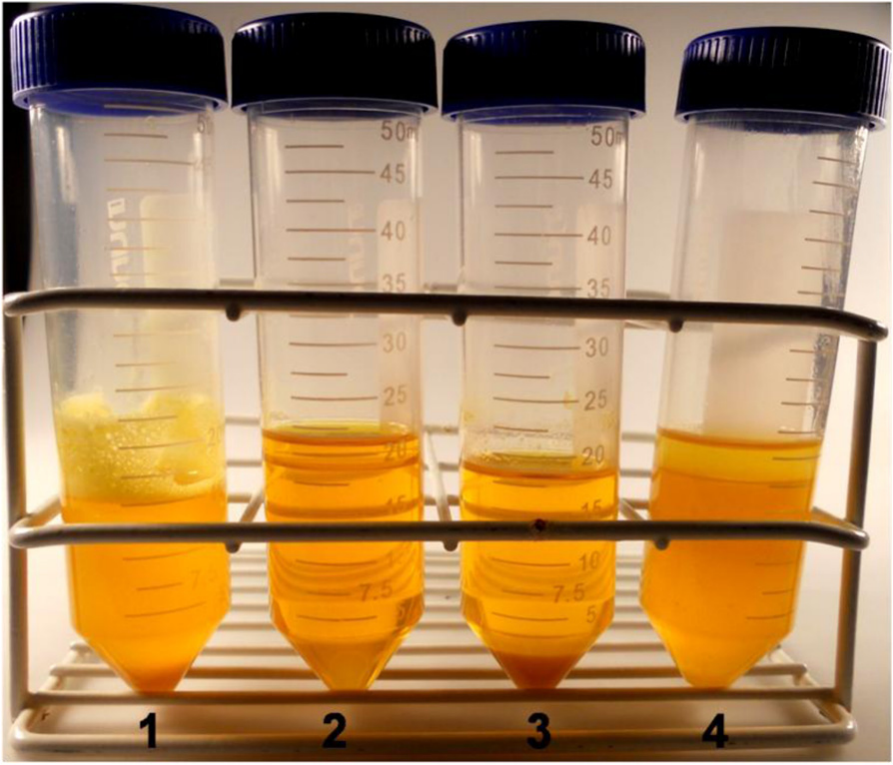
**Dissolving effect of methanol on oil gums. (1)** Soybean oil (raw material); **(2)** soybean oil with 1.5 eqs MeOH; **(3)** soybean oil acid degummed; and **(4)** soybean oil acid degummed with 1.5 eqs MeOH. Image obtained after 24 h incubation at 35°C, 250 rpm agitation, and centrifugation at 2,000 rpm for 5 min.

In order to better see the effect of methanol on phospholipids and to investigate the combined action of citric acid and methanol, these assays were repeated using excess methanol (1.5 eqs), added to the simple raw material and to citric acid-treated oil. The corresponding reaction mixtures appeared as shown in Figure 2 after centrifugation. Comparison of batches 1 (no methanol) and 2 (with methanol) containing untreated raw material clearly reflects the effect of methanol on gums removal. When methanol was present (batch 2) the oil phase was completely clear and no sediment could be observed. Oil clarification was also observed when citric acid degumming was applied (batch 3), but in this case a sediment formation appeared, suggesting gums precipitations. Batch 4, containing both citric acid and methanol in the reaction mixture, displayed a non-transparent oil phase resulting probably from a contrasting effect on phospholipids solubility due to the double treatment. From the results obtained, and in agreement with previous reports derived from other studies, we can conclude that methanol seems to solubilize the phospholipids of soybean oil [25]. Furthermore, soybean oil phospholipids are mainly constituted by PC (and to a lower extent by PE), described as easily hydratable phospholipids and also soluble in short chain alcohols like ethanol [26]. Thus, their solubility in methanol would also be possible and is in agreement with the results obtained here.

Given the widespread use of soybean oil in industrial biodiesel production, the evidence of solubilization of phospholipids by methanol acquires a great importance in the process. It means that gums can be dissolved by methanol, with no requirement for a conventional acid treatment, thus making the released phospholipids more available for enzymatic transesterification, that is, methanol seems to break apart the micelles. Therefore, presence of methanol, used here as a substrate, may allow to abolish the acid degumming step without any loss of performance. This assumption was demonstrated with the combination of enzymatic degumming and transesterification, performed without citric acid treatment, as described in the following sections.

### Combining enzymatic degumming and transesterification

Oil degumming is a requirement to obtain refined, edible oils, but it is also essential for biodiesel production. For immobilized *Candida antarctica* lipase, Watanabe and co-workers reported that crude (non-degummed) oil does not undergo enzymatic-catalyzed methanolysis [6]. Depending on the raw materials used, degumming becomes an indispensable step for biodiesel production to achieve phospholipids removal and to reduce the final phosphorus content below the specified limits. In addition to an extra tank, the degumming process involves the use of acids and high temperatures, all factors boosting the process costs. Moreover, during the degumming process there is an unavoidable loss of oil that migrates to the gums during removal. For instance, for crude soybean oil containing an average 900 ppm P, gums represent a 2.5% loss of total oil; being the current market price US$1,100 per ton, this corresponds to a loss of US$27.5 per ton of oil. These drawbacks could be overcome with the unification of degumming and transesterification in the same tank [18]. For this purpose, a single-step enzymatic degumming and transesterification process using phospholipases and liquid lipase Callera Trans L, with no need for a conventional acid degumming treatment, could provide a solution to such problems.

**Figure 3:**
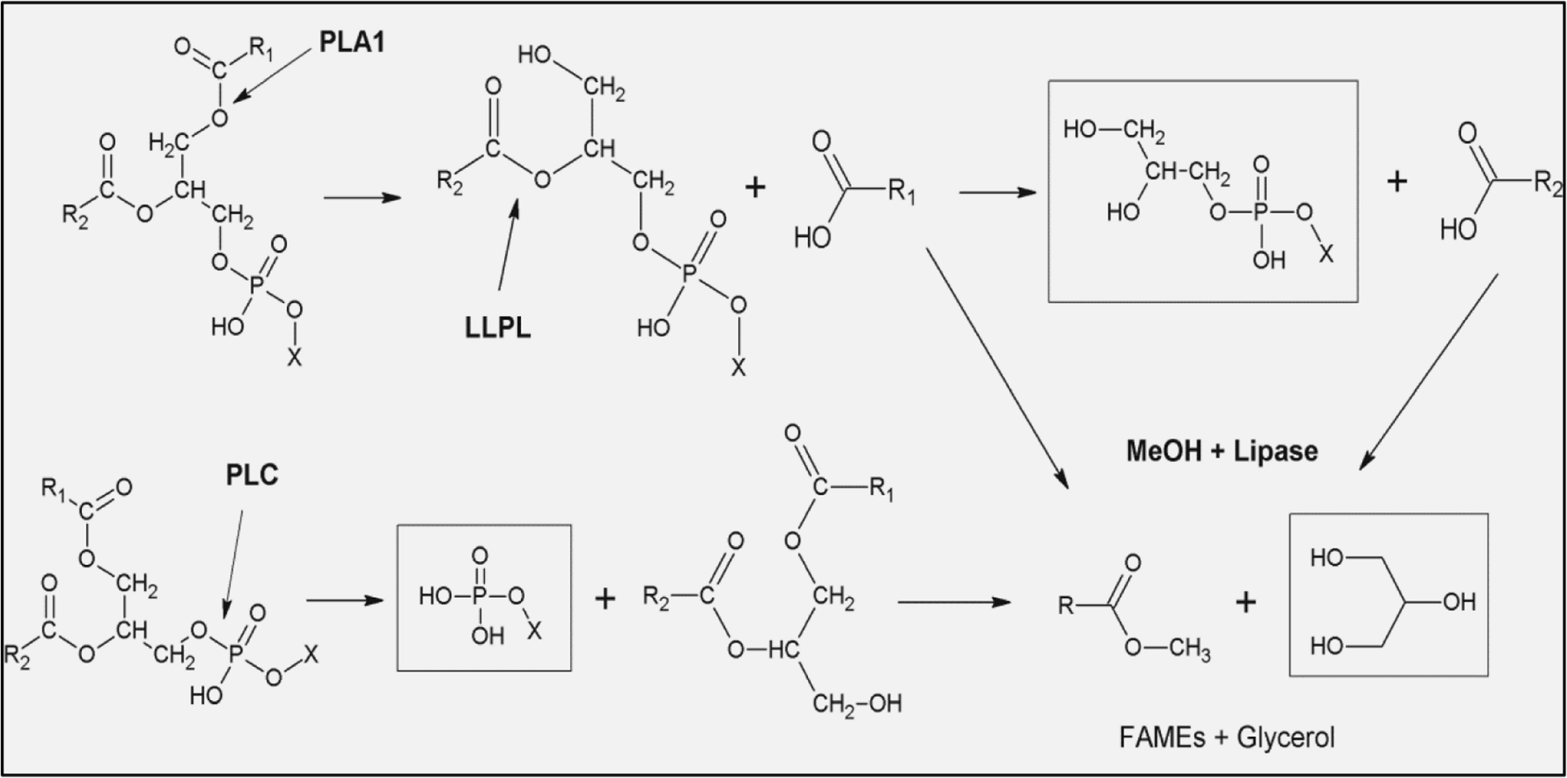
**Enzymatic activities in the combined process.** Schematic representation of all possible enzymatic activities involved in the combined degumming/transesterification process. Polar glycerol-phosphatide, resulting from PLA_1_ + LLPL-2 activities, and the phosphodiester group resulting from PLC cleavage, migrate to the aqueous phase together with the glycerine produced during transesterification (polar compounds highlighted by rectangles). LLPL-2, lyso-phospholipase; PLA_1_, phospholipase A_1_; PLC, phospholipase C.

Since citric acid has a negative effect on FAMEs production (Figure 1), and taking into consideration the role of methanol in phospholipid solubilization shown above (Figure 2), enzymatic degumming with phospholipases was coupled to transesterification in the same batch, using Callera Trans L best operating conditions (24 h incubation, 35°C, 250 rpm). Even though in some cases, as for PLC, optimal pH and temperature conditions were different from those of Callera Trans L (Table 1), the prolonged reaction time could compensate for the slower catalysis rate of such phospholipase. Performance of different types of phospholipases was tested and the final results for FAMEs production and phosphorus content are listed in Table 4. From the point of view of final phosphorus content, it is remarkable the high P concentration found in the oil phase when only transesterification (TE), used as a control, was run. For this sample, after the reaction and recovery by mild centrifugation, the phosphorus content was approximately the same as that of raw material (823 ppm), suggesting that Callera Trans L does not have hydrolytic phospholipase activity. Moreover, FAMEs formation in that case was not complete, with only 85% FAMEs production achieved. The transesterification yield increased drastically, to reach >95% FAMEs, when phospholipases were applied to the reaction mixture, especially when PLA_1_ was present (Table 4). At the same time, a dramatic phosphorus content decrease, below 10 ppm, was found in all reaction mixtures including phospholipases. The only exceptions were those reactions containing solely PLC and Callera Trans L. For those samples FAMEs production resulted in approximately 90%, and remaining phosphorus was 12 ppm. The highest FAMEs production in samples containing combinations of phospholipases could be explained by a synergic effect between phospholipases and the lipase during the process. PLA_1_ would first release a FFA from a phospholipid molecule, making it available for lipase esterification with methanol, a fact that has already been reported for Callera Trans L, which shows an excellent esterification activity [11]. Therefore, through a synergic mechanism, phospholipase activity leads to a gain of oil useful for lipase-mediated esterification. Thus, beyond the reduction of phosphorus in the final biodiesel, phospholipases contribute to the release of FFAs from phospholipid molecules, which can then be used by the lipase for FAMEs formation. Moreover, when a lyso-phospholipase (LLPL-2) was added to help PLA_1_ in reducing the final content of phospholipids, the phosphorus values found were even lower. This can be explained by the mode of action of LLPL-2, which releases the FFA located at position 2 left by PLA_1_ activity on the phospholipid molecule. After the combined action of PLA_1_ and LLPL-2, a glycerol-phosphatide is liberated (Figure 3) which, being more polar than a lyso-phospholipid, migrates to the glycerol phase, thus reducing the phosphorus content in the oil phase. Higher reductions of phosphorus could be observed when PLA_1_ was combined with PLC. PLC directly cuts the phosphodiester group, releasing a DAG (Figure 3), also known to be a good substrate for Callera Trans L [11]. FAMEs production and phosphorus reduction when only PLC was applied resulted in values lower than in other reactions, a fact that can be justified by the selectivity of this enzyme for only two kinds of phospholipids (PC and PE) [27]. Furthermore, PLC was the enzyme working in the worst conditions in the combined process, as it was used far from its optimum temperature. Despite that, we can point to PLC as an ideal helper of PLA_1_ in the combined process developed. Figure 3 graphically represents the enzyme activities mentioned and summarizes the polarity of the species released. Among them, glycerol and polar molecules containing the remaining phosphorus, like the glycerol-phosphatide and the phosphodiester group (enclosed in rectangles), are shown, which are easily separated into the glycerin phase. This explains the good reduction of phosphorus content achieved in the final biodiesel for samples treated with the enzyme combinations assayed. The proposed activity of the phospholipases tested and their released products was further confirmed after study of the phosphatides composition of the oil and glycerin phases resulting from the combined reactions performed.

**Table 4:** Biodiesel resulting from the combined degumming/transesterification process

**Condition**	**FAMEs**^**a**^	**P**^**b**^
TE	85.2 ± 1.4	823 ± 56
PLA_1_ + TE	98.2 ± 2.1	8.0 ± 3.0
PLC + TE	90.8 ± 4.0	12.8 ± 1.8
PLA_1_ + LLPL-2 + TE	97.8 ± 0.3	6.0 ± 3.0
PLA_1_ + PLC + TE	96.6 ± 0.8	4.6 ± 1.7

^a^FAMEs production measured as percentage; ^b^phosphorus content expressed in ppm. All data are the mean of three different trials. FAME, fatty acid methyl ester; LLPL-2, lyso-phospholipase; PLA_1_, phospholipase A_1_; PLC, phospholipase C; TE, transesterification.

### Study of phospholipases activity

Analysis of phosphatides by ultra-performance liquid chromatography tandem mass spectrometry (UPLC/MS/MS) in the oil and glycerin phases obtained from the combined reactions, confirmed the enzymatic activity of phospholipases, excluding the possibility that the phospholipids present in the original raw material were separated to the glycerin phase or to the interphase as entire molecules, without any gain of oil for further lipase-catalyzed transesterifications.

**Figure 4:**
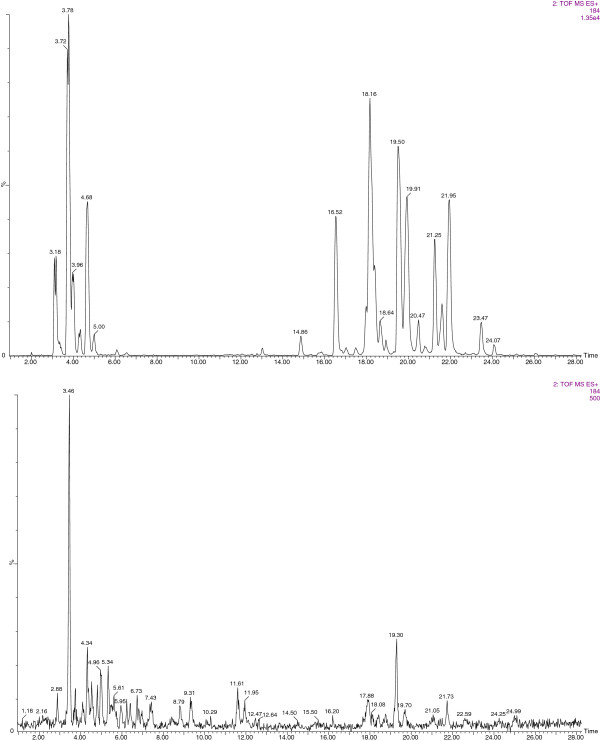
**UPLC/MS/MS analysis of the oil phase.** Comparison of the mass spectrum resulting from analysis of the oil phase of reactions TE (upper plot) and PLA_1_ + TE (bottom plot). Samples were run with a RP column. For both chromatograms, ion 184 m/z in positive MS/MS mode were extracted. MS/MS, tandem mass spectrometry; PLA_1_, phospholipase A_1_; RP, reverse phase; TE, transesterification; UPLC, ultra-performance liquid chromatography.

Comparing the oil phase of control samples containing only Callera Trans L (TE), where only transesterification occurs, with that resulting from reactions containing both the lipase and PLA_1_ (PLA_1_ + TE), it can be observed that peaks corresponding to intact phospholipids containing PC (RT = 15 to 24 min region) completely disappeared (Figure 4), indicating that PLA_1_ activity almost completely removed all PC from the oil phase. Interestingly, peaks corresponding to LPC (RT = 3 to 5 min region), apparently present in the oil from the beginning, disappeared after PLA_1_ + TE treatment, suggesting a kind of extra activity of PLA_1_, which in this case seems to be able to process also the fatty acid of the LPC molecule and not only the one at position sn-1 as would be expected. This extra activity, not described before for a PLA_1_, could be due to the long reaction time used for the combined process: generally phospholipases are used for 2 to 6 h (Table 1), while in our system, they were acting during 24 h.

The final glycerin composition of all samples was analyzed for each type of phosphorous compound, and the mode of action of the phospholipases used could be confirmed (see Additional files 1, 2, 3, 4, and 5). As expected, PC (RT = 5 min; m/z 184) could be found only in the glycerin phase derived from the reactions where PLC was present (PLC + TE and PLA_1_ + PLC + TE), proving the specific activity of this phospholipase for the phosphodiester bond (see Additional file 3 and Additional file 5). Instead, as a result of PLA_1_ activity, lyso-phosphocholine should appear at RT = 1.88 min, extracting ions 496.3, 518.3, 520.3, and 522.3 m/z (corresponding to LPC bearing the 16:0, 18:1, 18:2, or 18:3 fatty acids, respectively), but it could also be detected in very small amounts in all glycerin samples, suggesting a kind of instability of the LPC molecule. In fact, looking at glycerol-phosphocholine (RT = 11 min; m/z 258), this product should be present only in the glycerin phase of the reaction PLA_1_ + LLPL-2 + TE, resulting from the combined activity of these enzymes. Nevertheless, it was also found in all other samples, supporting the hypothesis of an extra activity of PLA_1_ in processing the fatty acid at position sn-2, or being the consequence of a kind of instability of the LPC molecule (see Additional files 1, 2, 3, 4, and 5). However, LLPL-2 activity could be confirmed by the peak of glycerol-phosphocholine found in sample PLA_1_ + LLPL-2 + TE, which was in fact higher than that found for the other samples (see Additional file 4). From these results we can conclude that parallel analysis by UPLC/MS/MS of the phosphatides found in the oil and glycerin phases, resulting from the combined reactions performed, turned out as a powerful tool for the study of such a complex system, and allowed to isolate the activity of each enzyme applied, confirming the importance of phospholipases in the combined degumming and transesterification process.

### Combined degumming/transesterification process using crude, difficult vegetable oils

The corn oil used here derived from a biorefinery-bioethanol plant production, and is obtained directly from dry corn milling during the ethanol process. The oil is isolated after fermentation and centrifugation, and is not degummed in the traditional sense, although it is somehow physically and chemically degummed throughout the ethanol process. This oil has a phosphorous content of 62 ppm, mostly constituted by hydratable phospholipids. Used as a substrate for combined enzymatic degumming and transesterification, it resulted in a slightly lower FAMEs production, compared to soybean oil. Optimum operational conditions for such corn oil were obtained with the combination PLA_1_ + LLPL-2 + TE, showing 96.2% release of FAMEs. Transesterification with corn oil could not be complete due to the composition of this substrate, rich in sterols, acylglycerides, and waxes, which probably are not processed by the lipase for methyl esterification. Nevertheless, the rate of FAMEs production with this oil is still >95%, as required by the biodiesel specifications. Like for soybean oil, phospholipases helped to increase the transesterification yield, as shown in Table 5. For all conditions tested, the final phosphorus values obtained after reactions were below 10 ppm, even when phospholipases were not present in the reaction mixture. This is mostly due to the low initial content of phosphorus in the corn oil and indicates that such phospholipids are mostly hydratable and easily removed by simple washing with the water present in the system. However, when a phospholipase is present in the reaction mixture, phospholipids are hydrolyzed and more useful oil is available for lipase methyl ester formation.

**Table 5:** Combined degumming/transesterification biodiesel process applied to difficult oils

	**Corn Oil**		**Canola oil**	
**Condition**	**FAMEs**^**a**^	**P**^**b**^	**FAMEs**^**a**^	**P**^**b**^
TE	93.4	7.5	98.7	145
PLA_1_ + TE	94.5	7.3	98.3	142
PLC + TE	95.7	9.2	98.2	130
PLA_1_ + LLPL-2 + TE	96.2	9.3	98.9	145
PLA_1_ + PLC + TE	95.5	7.5	99.1	130

^a^FAMEs production measured as percentage; ^b^final phosphorus content expressed in ppm. FAME, fatty acid methyl ester; LLPL-2, lyso-phospholipase; PLA_1_, phospholipase A_1_; PLC, phospholipase C; TE, transesterification.

When crude canola oil was used, a high transesterification rate was achieved but the final phosphorus content was not reduced below 130 ppm (Table 5). This oil is known to have a very high content of Ca^2+^ and Mg^2+^, and is especially difficult to degum. Crude canola oil is considered one of the most difficult oils for degumming because it is mostly composed by NHPs. These NHPs are barely attacked by phospholipases without the aid of an acid treatment. Moreover, they need a strong chelating agent such as citric acid at higher concentrations (0.1%) to achieve a suitable phosphorus reduction. These conditions can hardly be reached with milder conditions of citric acid or even with phosphoric acid (D Cowan, personal communication), thus making it difficult to reach the required phosphorus content in the final biodiesel.

## Conclusions

A successful, completely enzymatic process has been investigated resulting in a more economic and eco-friendly biodiesel production. Combination of crude oil degumming and transesterification in a unique step is possible by using phospholipases and liquid lipase Callera Trans L. In the combined process, an important cost reduction can be achieved. In addition to the US$27.5 per ton savings in the case of soybean oil, costs can be substantially lowered by avoiding the extra tank commonly required for oil degumming pretreatments, and by using mild temperatures (35°C). Moreover, citric acid treatment has been eliminated and only low sodium hydroxide concentrations are used, thus increasing the savings of the whole process. Therefore, the developed method meets the conditions for being easily scaled-up and is suitable for most crude vegetable oils.

## Methods

### Substrates

Crude soybean oil (FFA = 1%; P = 900 ppm; pH = 6.8) was obtained from Cargill (Iowa Falls, IA, USA). Corn oil from bioethanol production (FFA = 6%; P = 62 ppm; pH = 4.5) was provided by Blue Sun (St Joseph, MO, USA). Crude canola oil (FFA = 1%; P = 250 ppm; pH = 5.7) was kindly donated by Richardson (Winnipeg, MB, Canada). Crude soybean oil was chosen as a cheaper raw material and because it is still the most commonly used oil in the industrial biodiesel production. Corn oil was considered interesting for this study because it derives, as a residue, from the bioethanol industrial production, thus to close the hypothesis to unify both bioethanol/biodiesel production processes. Finally, crude canola oil was tested to verify the potentialities of the suggested process towards oils rich in NHPs; the canola oil used here contained approximately 130 ppm of P in NHPs over the total 250 ppm of initial P content.

### Enzymes and chemicals

Soluble lipase Callera Trans L, phospholipase A_1_, PLA_1_ (Lecitase® Ultra), and lyso-phospholipase, LLPL-2 (patent WO 2001027251 A1) used in this work were from Novozymes A/S. Phospholipase C, PLC (Purifine®) was purchased from Verenium (San Diego, CA, USA). All chemicals used were from Sigma Aldrich (St Louis, MO, USA).

### Enzymatic degumming

When only enzymatic degumming was performed, an adaptation of the Cowan and Nielsen protocol was used]. Acid treatment was performed by adding citric acid (0.065%) to 20 g of pre-heated oil (55°C) and mixed with an Ultra Turrax® T25 (IKA, Staufen, Germany) for 10 s at 12,000 rpm. The emulsion was incubated for 30 min at 55°C and 250 rpm in a horizontal shaker. Caustic neutralization was performed with addition of NaOH (2 eqs to citric acid) and 3.5% water. Enzymatic degumming was completed by applying phospholipases, with the recommended dosage indicated in Table 1. Lyso-phospholipase was used only in combination with PLA_1_ at a concentration of 400 ppm. Combined degumming/transesterification reactions were run at 35°C for 24 h with 250 rpm agitation, according to the optimum transesterification conditions, instead of the recommended incubation for degumming of 55°C for 2 h.

### Transesterification reactions

FAMEs synthesis reactions were carried out in 100 ml squared bottles for 24 h at 35°C with 250 rpm agitation in a horizontal shaker. The reaction mixtures consisted of 20 g oil, 1% w/w Callera Trans L lipase solution, 3.5% H_2_O, and 10 ppm of NaOH. Total methanol (MeOH) per reaction was 1.5 eqs of oil, added continuously by a syringe pump system (SP220 IZ, WPI) with a flow rate of 0.4 ml/h during 10 h.

### One-step enzymatic degumming and transesterification

All enzymes (lipase and phospholipases) were added at a time to 20 g of oil. Reaction mixtures included water (3.5%), NaOH (10 ppm), and 1.5 eqs of MeOH, pumped in a linear gradient for 10 h. Combination of degumming and transesterification was incubated following the transesterification conditions.

### Statistical design of experiments

The effect of citric acid was studied by a RSM, where two variables were analyzed at three different levels: 1) choice of acid (citric/phosphoric); 2) the equivalents of NaOH, necessary to balance the pH (1 to 1.5 to 2 eqs); and 3) extra NaOH, generally helpful for transesterification of difficult oils (0 to 10 to 20 ppm). These conditions were combined with the citric/phosphoric acid possibility. Distribution of the experimental patterns analyzed is shown in Table 2. Each pattern corresponds to a single batch reaction, where the acid treatment, followed by caustic neutralization, was combined directly with the transesterification without any phospholipase addition. Citric acid, at a fixed concentration of 0.065% w/w of oil, or phosphoric acid at 0.025% w/w, were added to 20 g of oil, mixed by high shearing and incubated for 30 min at 55°C, with 250 rpm agitation. After incubation, the oil was cooled down and Callera Trans L (1% w/w) was added together with 3.5% water and NaOH, as indicated in Table 2. Incubation was prolonged for 24 h at 35°C with a linear gradient pumping of methanol (0.4 ml/h for 10 h). Each pattern was analyzed for FAMEs production and final phosphorus content. Data were analyzed with JMP software (SAS Institute Inc.).

### Recovery methods

Analysis of FAMEs production was performed by gas chromatography (GC). After 24 h incubation, 1 ml reaction mixture was taken and evaporated in a Heto Vacuum concentrator at 60°C for 1½ h to remove excess methanol.

For phosphorus analysis, the whole reaction volume was transferred to a 50 ml tube and centrifuged at low speed (2,000 rpm) for 5 min to simulate the sedimentation step used to separate the final products in an industrial production plant. After centrifugation, 4 ml were taken from the upper oil phase and analyzed through ICP-OES. The bottom phase (glycerin) was analyzed by UPLC/MS/MS to study the phosphatides composition resulting from the reactions.

### FAMEs determination

Determination of FAMEs (%) was performed according to the EN14103 standard method on a Varian Chrompack CP-3900 GC with flame ionization detectors (FIDs), equipped with a Varian ‘Select Biodiesel for FAMEs’ (30 m, 0.32 id) column. Methyl heptadecanoate was used as internal standard, as indicated by EN14103. The solution was prepared at a concentration of 10 mg/ml in acetone. After methanol evaporation, 50 mg of the oil phase were used for each analysis.

### Phosphorus content quantification

Phosphorus content was determined by the ICP-OES method at the department of Analytical Development, Novozymes (Kalundborg, Denmark). Accordingly, 0.2 g of each sample were initially destructed in 4.5 ml concentrated HNO_3_ (69%) and heated for 4 to 5 h at 105°C for further dissolution in a total volume of 10 ml Milli-Q water. Treated samples were analyzed in a Varian Vista MPX system for ICP, using yttrium as internal standard. Resulting data were processed with ICPExpert version 4.1 software, and phosphorus concentration expressed in ppm = mg/kg.

### Analysis of phosphatides

Phosphatides content in the oil and glycerin phase was analyzed by UPLC/MS/MS in a Q-Tof Premier (Waters, Milford, MA, USA). Two chromatographic systems were set up: one with a hydrophilic interaction liquid chromatography (HILIC) column (Acquity BEH Amide, 1.7 μm, 2.1 mm × 150 mm), and the second with a reverse phase (RP) column (Acquity CSH C18, 1.7 μm, 2.1 mm × 100 mm). Samples were analyzed in positive and negative mode on the UPLC-UV-Tof and data processed using MassLynx version 4.1 software (Waters). The RP-chromatography was set up with a 0.25 ml/min flow of eluent A containing acetonitrile/ isopropyl alcohol/HCOOH (50:50:0.15) and eluent B containing isopropyl alcohol/HCOOH (100:0.15). The gradient was running isocratic for 1 min at 99% A, followed by a 49 min gradient to 1% A. The 1% A was running isocratic for 5 min, followed by a 5 min gradient back to initial settings (that is, 99% A). The HILIC was set up with a 0.35 ml/min flow and an A-eluent with acetonitrile/HCOOH (100:0.15%) and B-eluent with acetonitrile/MQ water/HCOOH (50:50:0.15). The method was running isocratic for 20 min with 95% A. For both procedures, the MS was set to scan from 95 to 1,500 m/z ions in both positive and negative mode.

Commercial phosphorous compounds of choline (LPC, glycerol-phosphocholine, and PC) were chosen as standards and run in the UPLC/MS/MS with the HILIC column. Corresponding retention times and mass ion compositions are listed in Table 6. Glycerin samples analyzed were the same as those shown in Table 4 for the combined degumming and transesterification reactions using soybean oil.

**Table 6:** UPLC/MS/MS analysis of phosphatide compounds used as standards

**Compound**	**RT (min)**	**Ion m/z**
Lyso-phosphocholine	1.88	^a^518
		520
		522
Glycerol-phosphocholine	11	258
Phosphocholine	5	184

^a^Ions corresponding to molecules containing a 18:1, 18:2, and 18:3 fatty acid chain, respectively. RT, retention time; UPLC/MS/MS, ultra-performance liquid chromatography tandem mass spectrometry.

## Abbreviations

AD: Acid degumming; DAG: Diacylglycerol; FAME: Fatty acid methyl ester; FFA: Free fatty acid; FID: Flame ionization detector; GC: Gas chromatography; HILIC: Hydrophilic interaction liquid chromatography; ICP-OES: Inductively coupled plasma optical emission spectrometry; LLPL: Lyso-phospholipase; LPC: Lysophosphatidylcholine; MeOH: Methanol; MS/MS: Tandem mass spectrometry; NHP: Non-hydratable phospholipid; PA: Phosphatidic acid; PC: Phosphocholine; PE: Phosphatidylethanolamine; PI: Phosphatidylinositol; PLA1: Phospholipase A_1_; PLC: Phospholipase C; RP: Reverse phase; RSM: Response surface methodology; RT: Retention time; TE: Transesterification; UPLC: Ultra-performance liquid chromatography.

## Competing interests

The authors declare that they have no competing interests.

## Authors’ contributions

SC participated in the design of the study, carried out the experiments, organized and interpreted the data, and drafted the manuscript. RFH performed the ICP-OES and UPLC/MS/MS analysis, and contributed to the manuscript draft. PD contributed to critical discussion, and revised and corrected the manuscript. PMN coordinated the project and the design of the study, critically interpreted the data, and revised the manuscript. All authors read and approved the final manuscript.

## Supplementary Material

Additional file 1 **UPLC/MS/MS analysis of phosphorous compounds found in the released glycerin after a degumming/transesterification reaction.** Positive MS spectrum corresponding to TE sample, where only Callera Trans L was present. LPC, glycerol-phosphocholine, and PC were analyzed by extracting ions 520, 258, and 184 m/z, respectively. Click here for file

Additional file 2 **UPLC/MS/MS analysis of phosphorous compounds found in the released glycerin after a degumming/transesterification reaction.** Positive MS spectrum corresponding to PLA_1_ + TE sample, where PLA_1_ and liquid lipase Callera Trans L were present. LPC, glycerol-phosphocholine, and PC were analyzed by extracting ions 520, 258, and 184 m/z, respectively. Click here for file

Additional file 3 **UPLC/MS/MS analysis of phosphorous compounds found in the released glycerin after a degumming/transesterification reaction.** Positive MS spectrum corresponding to PLC + TE sample, where PLC and liquid lipase Callera Trans L were present. LPC, glycerol-phosphocholine, and PC were analyzed by extracting ions 520, 258, and 184 m/z, respectively. Click here for file

Additional file 4 **UPLC/MS/MS analysis of phosphorous compounds found in the released glycerin after a degumming/transesterification reaction.** Positive MS spectrum corresponding to PLA_1_ + LLPL-2 + TE sample, where PLA_1_, lyso-phospholipase 2, and liquid lipase Callera Trans L were present. LPC, glycerol-phosphocholine, and PC were analyzed by extracting ions 520, 258, and 184 m/z, respectively. Click here for file

Additional file 5 **UPLC/MS/MS analysis of phosphorous compounds found in the released glycerin after a degumming/transesterification reaction.** Positive MS spectrum corresponding to PLA_1_ + PLC + TE sample, where PLA_1,_ PLC, and liquid lipase Callera Trans L were present. LPC, glycerol-phosphocholine, and PC were analyzed by extracting ions 520, 258, and 184 m/z, respectively. Click here for file
