# Supplementary material for: Combining phospholipases and a liquid lipase for one-step biodiesel production using crude oils
Source: Biotechnol Biofuels. 2014 Feb 26;7:29. doi: 10.1186/1754-6834-7-29 (PMC4015511; doi:10.1186/1754-6834-7-29)
Supplement: Additional file 1 — UPLC/MS/MS analysis of phosphorous compounds found in the released glycerin after a degumming/transesterification reaction. Positive MS spectrum corresponding to TE sample, where only Callera Trans L was present. LPC, glycerol-phosphocholine, and PC were analyzed by extracting ions 520, 258, and 184 m/z, respectively. [file 1754-6834-7-29-S1.docx]

**Additional file 1.docx**: **UPLC/MSMS analysis of phosphorous compounds found in the released glycerin after a degumming/transesterification reaction.** Positive MS spectrum corresponding to TE sample, where only Callera Trans L was present. LPC, glycerol-phosphocholine and phosphocholine were analyzed by extracting ions 520, 258 and 184 m/z, respectively.
